# Urinary proteomic shotgun approach for identification of potential acute rejection biomarkers in renal transplant recipients

**DOI:** 10.1186/2047-1440-1-9

**Published:** 2012-08-31

**Authors:** Håvard Loftheim, Karsten Midtvedt, Anders Hartmann, Anna V Reisæter, Pål Falck, Hallvard Holdaas, Trond Jenssen, Leon Reubsaet, Anders Åsberg

**Affiliations:** 1 Department of Pharmaceutical Chemistry, School of Pharmacy, University of Oslo, P.O.Box. 1068, Blindern, NO-0316, Oslo, Norway; 2 Department of Pharmaceutical Biosciences, School of Pharmacy, University of Oslo, Oslo, Norway; 3 Department of Transplant Medicine, Oslo University Hospital, Rikshospitalet, Norway

**Keywords:** Acute rejection, Biomarker, Renal transplantation, Urinary proteomics

## Abstract

**Background:**

Acute rejection (AR) episodes in renal transplant recipients are suspected when plasma creatinine is elevated and other potential causes out ruled. Graft biopsies are however needed for definite diagnosis. Non-invasive AR-biomarkers is an unmet clinical need. The urinary proteome is an interesting source in the search for such a biomarker in this population.

**Methods:**

In this proof of principle study, serial urine samples in the early post transplant phase from 6 patients with biopsy verified acute rejections and 6 age-matched controls without clinical signs of rejection were analyzed by shotgun proteomics.

**Results:**

Eleven proteins fulfilled predefined criteria for regulation in association with AR. They presented detectable regulation already several days before clinical suspicion of AR (increased plasma creatinine). The regulated proteins could be grouped by their biological function; proteins related to growth and proteins related to immune response. Growth-related proteins (IGFBP7, Vasorin, EGF and Galectin-3-binding protein) were significantly up-regulated in association with AR (*P* = 0.03) while proteins related to immune response (MASP2, C3, CD59, Ceruloplasmin, PiGR and CD74) tended to be up-regulated ( *P* = 0.13).

**Conclusion:**

The use of shotgun proteomics provides a robust and sensitive method for identification of potentially predictive urinary biomarkers of AR. Further validation of the current findings is needed to establish their potential clinical role with regards to clinical AR diagnosis.

**Trial registration:**

ClinicalTrials.gov number NCT00139009

## Background

Patients whom experience an acute rejection (AR) after renal transplantation have an increased risk of developing chronic allograft nephropathy and reduced long-term graft survival [1-5]. In a clinical setting an AR is typically suspected upon an increase in plasma creatinine that cannot be explained by other plausible causes, and verified by histological examination of core biopsies from the graft [6]. This method is however flawed by both late and unspecific onset of plasma creatinine increase and sampling heterogeneity and poor correlation with treatment response and prognosis for biopsy results.

Even though renal biopsying *per se* is considered a relatively safe procedure when appropriate clinical precautions are taken, it is a time-consuming invasive procedure which is cumbersome for the patients and has potential side effects [7]. In the general follow-up of transplanted patients a non-invasive method with high sensitivity and specificity for diagnosing AR is desirable. Of the many different methods and matrices plausible for such monitoring, the urinary proteome is maybe one of the most appropriate. It can be accessed non-invasively and the proteome reflects the last step in molecular regulation of immune responses. About 30% of the urinary proteome comes from plasma while the rest is locally produced in the kidney, increasing the possibility of reflecting kidney specific processes [8,9]. This is likely an advantage when monitoring graft function and events in kidney transplantation. Several attempts have been made to identify possible urinary biomarkers for AR [10-24], but none are currently used clinically [25,26]. Most of the studies done are hypothesis based and only focus on a few specific target proteins. The development in the field of mass spectrometry has, however, made screening analysis of the full proteome technically possible. Recently, Sigdel *et al.* used shotgun proteomics to identify proteins in *pooled* urine samples from pediatric kidney transplants with acute rejection [20].

We performed a small prospective proof of principle study in order to show the applicability of using shotgun proteomics in serial samples from distinct individuals in the search for urinary proteins that are regulated in association with AR episodes. In shotgun proteomics proteins are enzymatically digested into peptides, which are separated by liquid chromatography, coupled to a mass spectrometer, in this case a state of the art LTQ-Orbitrap. This enables analysis of the whole proteome in one experiment utilizing the increased sensitivity offered by MS-detection of peptides instead of intact proteins. The very complex peptide mixture resulting from tryptic digestion of proteins requires more molecular information for unambiguous identification, which is achieved by the use of tandem mass spectrometry. After the first mass scan energy is added to the peptides, resulting in fragmentation and cleavage into amino acids which can be detected in the next mass scan allowing peptide sequencing and subsequent protein identification by database searches. In order to quantify protein levels in this method, samples were labeled with the stable ^18^O isotope and compared with respective baseline sample. The typical time-span of one single analysis is approximately 4–5 days, making this approach unsuitable for routine analysis. With this experimental setup the respective samples are mixed early in the process, acting as each other’s controls, which eliminates many of the factors contributing to experimental variability.

## Patients and methods

### Study design and samples

We used urine samples from 6 renal transplant patients with BPAR during the first post transplant months and from 6 renal transplant patients with stable graft function in the same period, matched for age, immunosuppression and time after transplantation. All urine samples were collected prospectively as part of an at that time ongoing study of twenty renal transplant recipients at Oslo University Hospital, Rikshospitalet [27]. On average urine samples were available from 4.7 ± 2.7 days after transplantation and the patients were followed for 8–10 weeks. All patients received induction with intravenous basiliximab on day 0 and 4, cyclosporine A (CsA), mycophenolate mofetil 1 g BID and steroids. Urinary samples were collected three times weekly the first two weeks, twice weekly the next four weeks followed by 1–2 samples per week thereafter. Acute rejections were suspected based on plasma creatinine increase (≥20%), after ruling out other potential causes such as bacterial infection or drug toxicity etc., and were verified by renal core biopsies (Banff 97 criteria) [6]. Urine samples from the day of BPAR were compared with the first available sample after transplantation (baseline) and from a clinically stable phase, approximately one week prior to rejection. In the control group no biopsies were obtained but urine samples from were attained at similar time points as in the AR-group.

The study was performed in accordance with the Declaration of Helsinki, local laws and regulations, including the Declaration of Helsinki and Declaration of Istanbul. The study was reviewed by the regional ethics committee and signed informed consent, covering also these urinary proteomics analyses, was obtained before study start from all patients. The trial EudraCT number is 2005-000219-90 and it is registered on http://www.clinicaltrials.gov (NCT00139009).

### Urine sample preparation

Midstream urine was collected without the addition of protease inhibitors and allowed to rest at 4°C for up to one hour, after which it was centrifuged at 800 × g for 10 minutes and stored at −70°C. Sample preparation was performed as previously described [28,29]. In short, total protein concentrations was measured using Bradford’s method [30] and the samples was normalized with respect to this, following cut-off filtration but prior to depletion. A volume of 300 μL was transferred to Vivapure Anti-HSA kit (Vivascience Sartorius Group) for albumin depletion. Reduction of the proteins was done using DTT at 95°C for 15 minutes, followed by alkylation with iodoacetic acid in the dark at room temperature for 15 min. Tryptic digestion and ^18^O/^16^O-labeling of the samples was done as described earlier [29]. The key parameters were as follows: A sample volume of 50 μL was applied to immobilized trypsin beads and digested using a pH 8.0 buffer at 37°C for 90 minutes under shaking (1200 rpm). Subsequently, the samples were subjected to ^18^O/^16^O-labeling using the same beads, but with a different buffer (pH 6.0) at 37°C for 3 hours under shaking (1200 rpm). Finally, the samples were purified and desalted by using in-house produced C18-tips prior to 2D LC-MS/MS analysis. The AR samples were labeled with ^18^O and mixed with both unlabeled baseline samples and unlabeled samples from a clinically stable phase (7–11 days prior to rejection) in the AR-group. In the control group, the time matched samples after transplantation was labeled and mixed with unlabeled baseline samples.

### 2D LC-MS/MS

Two-dimensional LC-MS/MS was used for separation and detection of the tryptic digested peptide mixture. Hydrophilic Interaction Liquid Chromatography (HILIC) was used as the first dimension of separation and was done exactly as described previously [28,29]. Fractions were collected every minute, in total 30 fractions per sample. All fractions were evaporated on a SpeedVac (Thermo) and reconstituted in 60 μL of 2% MeCN in 20 mM formic acid. The nanoLC-MS/MS analysis was done using 20 μL of reconstituted fractions as described earlier [29] but with a slightly modified HPLC setup: The reconstituted fractions were trapped on a C18 5 mm x 300 μm id Acclaim PepMap 100 (5 μm) enrichment column (Dionex). The loading mobile phase 20 mM formic acid and MeCN (98/2, *v/v*) was delivered at 10 μL/min for 4 minutes. The sample was transferred to a 150 × 0.075 mm id Acclaim PepMap 100 (pore size 100 Å and particle diameter 3 μm; Dionex) at 300 nL/min. The mobile phases consisted of A: 20 mM formic acid and MeCN (95/5, *v/v*) and B: 20 mM formic acid and MeCN (5/95, *v/v*). A linear gradient was run from 0% to 50% B in 60 minutes. Subsequently, the elution strength was increased to 100%.

The nanospray ionization (NSI) source was operated in the positive ionization mode (360 μm od × 20 μm id distal coated fused silica emitter, 10 μm id tip (New Objective, Woburn, MA, USA). Experiments were performed in two scan events; from *m/z* 300 to *m/z* 2000 in the FT-Orbitrap with resolution *R* = 30000 and a data dependent MS/MS with wide band activation carried out on the highest *m/z* value. The *m/z* values fragmented were dynamically excluded for 15 sec in order to fragment lower intensity *m/z* values. Helium gas was used to cause collision-induced fragmentation at 35% relative collision energy.

### Identification and selection of proteins

The acquired MS data were analyzed and processed using Proteome Discoverer 1.2 (Thermo) software. The raw files were analyzed in 2 search nodes, where the first search node was a SEQUEST™ [31] search against the FASTA file ipi.HUMAN.v3.76. Carboxymethyl (C) was set as constant modification while oxidation (M) and ^18^O (2) on the C-terminal were chosen as variable modifications. The peptide tolerance was set to 10 ppm while MS/MS tolerance was ±0.8 Da and 2 “missed cleavages” were allowed using trypsin as enzyme. A decoy database search was performed by searching against a database containing the reversed protein sequences with a strict target false discovery rate (FDR) of 0.01 and a relaxed FDR of 0.05. Grouping of proteins were enabled and only the top ranked peptide hits below the FDR threshold (< 0.05) were accepted. The heavy label was set to ^18^O (2) on the C-terminal, while the light channel contained no modifications. Only unique peptides were used for quantification and the labeled: unlabeled (^18^O:^16^O) ratios were adjusted against the protein median of all the quantified proteins in each patient.

Potentially clinically relevant regulation in this proof of principle study was defined as a fold change of ≥1 (log 2) between baseline and follow-up in at least three patients in the rejection group but excluding proteins with significantly higher average ratio in the control group and proteins more frequently up-regulated in the control group.

### Statistics

The nature of this kind of proof of principle studies makes relevant statistical analyses difficult. In attempt to provide a higher statistical power the regulated proteins were groups by their biological function. For the evaluation of the demographic data and comparison of the groups, the Mann–Whitney *U* test was used. A *P*-value of <0.05 was considered statistical significant and all analyses were performed by Minitab version 16.1 (Minitab Inc., Coventry, UK).

## Results

### Patient demographics

Demographic data of the six patients with acute rejection and six controls are shown in Table 1. The patients in the AR-group experienced biopsy proven acute rejection (BPAR) episodes on average 42 ± 27 days after transplantation, all C4d negative. No significant differences were present between the groups with respect to recipient age, HLA mismatch or donor age. The baseline urine samples were obtained 5.0 ± 3.6 and 4.3±1.8 days after transplantation in the AR- and control group, respectively.

**Table 1:** Demographic data at time of inclusion

	**All**	**No-rejection group**	**Rejection group**	***P*value**
Gender (male/female)	7/5	3/3	4/2	
Weight (kg)	75.7 ± 10.2	80.2 ± 11.1	71.3 ± 7.7	0.09
Age (years)	55.0 ± 12.2	59.5 ± 5.4	50.5 ± 15.8	0.26
HLA mismatch (A + B)	1.2 ± 0.9	1.0 ± 1.1	1.3 ± 0.8	0.47
HLA mismatch (DR)	1.2 ± 0.7	1.2 ± 0.8	1.2 ± 0.8	1.00
HLA mismatch (DQ)	0.5 ± 0.5	0.4 ± 0.5	0.5 ± 0.5	0.86
Serum creatinine^a^	143 ± 48	119 ± 55	168 ± 24	0.07
Age donor (years)	51.5 ± 10.8	49.0 ± 14.8	54.0 ± 4.6	0.52
Deceased donor (n)	11/12	6/6	5/6	

Data are means ± SD.

^a^at time of BPAR and matched time-points, respectively.

### Up-regulated proteins during AR episodes

**Figure 1:**
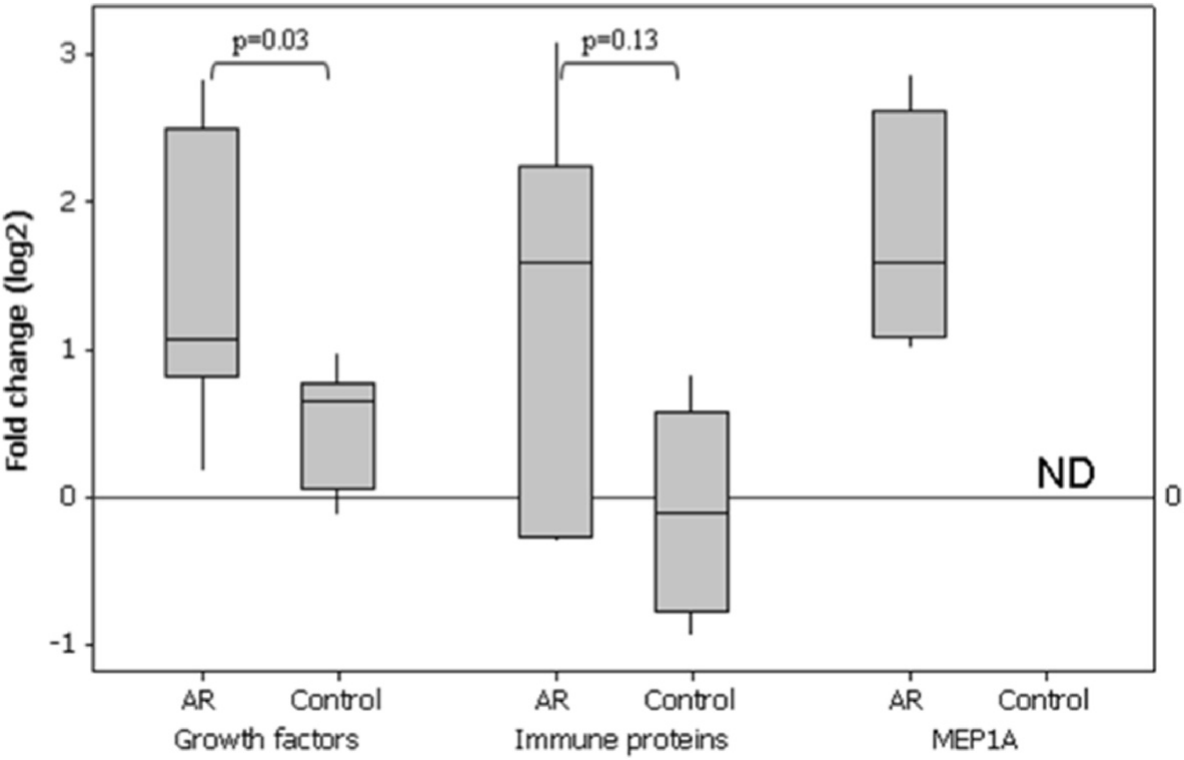
Box plot showing fold change (log2) of immune proteins, growth factors and MEP1A from baseline to acute rejection in the AR-group compared with the control group.

**Figure 2:**
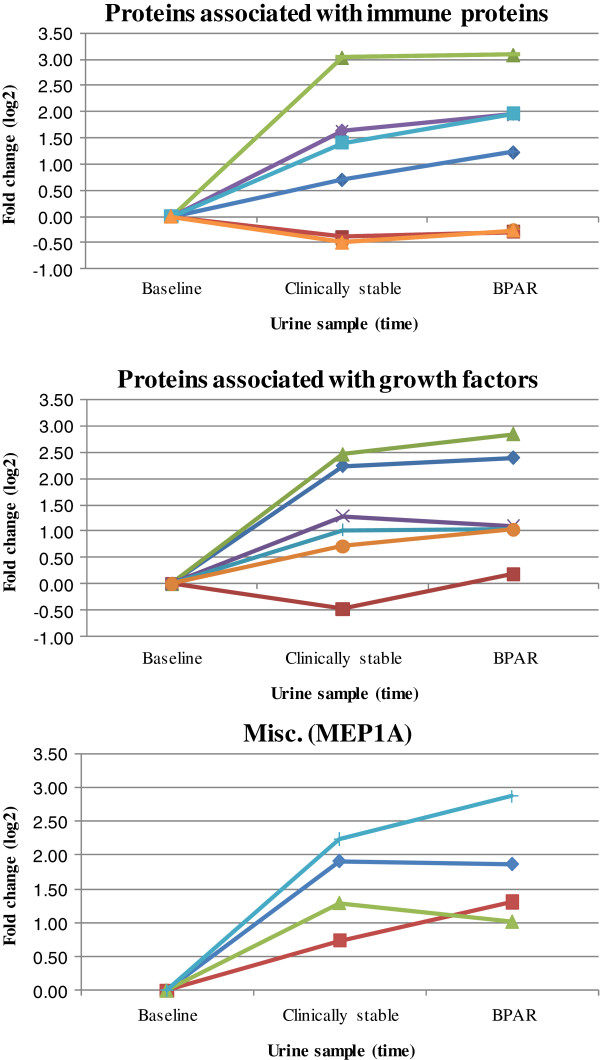
**Fold change (log2) of immune proteins, growth factors and MEP1A in the rejection group, AR1 (●) AR2 (×) AR3 (♦) AR4 (+) AR5 (■) AR6 (▴), from baseline to Biopsy Proven Acute Rejection (BPAR).** The center point (Clinically stable) is 7–11 days before BPAR, at stable serum creatinine levels.

A total of eleven proteins showed regulation according to the predefined criteria (Table 2). Ten of the proteins belonged to one of two main groups considering their biological function; proteins involved in regulation of growth and proteins involved in immune responses. Figure 1 presents a box plot of the eleven regulated proteins, grouped by biological function (average of ratios of the different proteins) and Meprin A subunit alpha (MEP1A), in the rejection group and in the controls. At the time of BPAR the growth factor proteins, as a group, were statistically significant up-regulated in the AR-group (*P* = 0.03). Five of six patients showed regulation of these proteins above the predefined threshold. A trend towards up-regulation was also present for the immune response proteins in the AR-group ( *P* = 0.13), present in four out of six patients, while none of the control patients showed regulation. MEP1A was not detected in any of the control patients but significantly up-regulated in all four AR-patients in which the protein was detected. Figure 2 shows the log 2 changes in protein levels for the specified protein groups between baseline and the time of BPAR in the AR-group. The trend is that these regulated proteins are up-regulated already in the clinically stable samples, 7–11 days prior to the time of BPAR.

**Table 2:** **Up-regulated proteins**^**a**^**in AR/control urine samples compared to baseline shown for individual patients**

			**Rejection group**						**No-rejection group**					
			AR 1	AR 2	AR 3	AR 4	AR 5	AR 6	C 1	C 2	C 3	C 4	C 5	C 6
		Banff classification	I_3_T_1_V_0_ C4d-	I_2_T_2_V_0_ C4d-	I_2_T_2_V_0_ C4d-	I_2_T_2_V_0_ C4d-	I_2_T_3_V_0_ C4d-	I_3_T_1_V_2_ C4d-	NA	NA	NA	NA	NA	NA
IPI ID	Gene ID	Protein name	**Log 2 change**						**Log 2 change**					
**Immune proteins**														
IPI00217775.1	CD74	Isoform 2 of HLA class II histocompatibility antigen gamma chain	ND^b^	0.47	**2.57**	**4.06**	**1.81**	**1.23**	0.39	**1.03**	0.81	0.99	**2.10**	0.58
IPI00004573.2	PIGR	Polymeric immunoglobulin receptor	−0.04	0.66	**2.27**	**1.31**	0.36	**2.20**	0.29	0.63	0.71	**1.65**	0.19	0.14
IPI00783987.2	C3	Complement C3 (Fragment)	−0.17	**3.17**	−0.48	**1.25**	**−5.32**	**2.64**	**−3.49**	ND	**1.14**	**−4.80**	**−1.54**	**−3.63**
IPI00017601.1	CP	Ceruloplasmin	0.42	**4.40**	0.34	**1.15**	ND	**4.79**	**−1.87**	**−1.25**	0.22	**−1.02**	0.53	**−2.02**
IPI00306378.5	MASP2	Isoform 2 of Mannan-binding lectin serine protease 2	**1.10**	**1.88**	**1.36**	**2.30**	**1.26**	**4.06**	0.57	**−3.94**	**1.94**	**1.19**	**2.44**	0.43
IPI00011302.1	CD59	CD59 glycoprotein	**−2.65**	**1.16**	**1.30**	**1.74**	0.40	**3.62**	**−1.52**	**2.68**	**−1.90**	**1.73**	**1.31**	0.19
**Growth factors**														
IPI00016915.1	IGFBP7	Insulin-like growth factor-binding protein 7	0.11	−0.43	**2.40**	**1.01**	−0.91	**2.84**	0.71	**1.42**	0.05	0.58	−0.02	ND
IPI00966866.1	EGF	Epidermal growth factor	**1.71**	**3.49**	**2.15**	0.75	0.97	ND	0.79	ND	ND	**1.51**	0.51	0.13
IPI00395488.2	VASN	Vasorin	**1.27**	**1.11**	ND	0.61	0.05	**2.10**	0.96	**−2.27**	**2.00**	0.66	**1.69**	−0.81
IPI00023673.1	LGALS3BP	Galectin-3-binding protein	**1.01**	0.20	**2.62**	**1.76**	0.61	**3.59**	0.26	**1.21**	0.03	**1.15**	0.32	0.30
**Other**														
IPI00004372.3	MEP1A	MEP1A protein (Meprin A subunit alpha)	ND	ND	**1.87**	**2.87**	**1.31**	**1.02**	ND	ND	ND	ND	ND	ND

^a^ Criteria for up-regulation: up-regulation (log 2 change ≥1) from baseline to AR in at least three patients in the AR-group. Proteins with higher average ratio in the control group and proteins more frequently up-regulated in the control group were excluded. ^b^ ND: Not detected.

## Discussion

The present analysis identified several up-regulated urinary proteins, but no relevantly down-regulated, in association with acute rejection episodes in the early post transplant phase after kidney transplantation. The results demonstrate the applicability of combining shotgun proteomics with relative quantification by ^18^O/^16^O-labeling in biomarker discovery using sequential samples from several patients. This labeling allows determining the relative amount of the proteins identified of two complex samples in one single analysis. Since sample handling is long, consist of many steps, is laborious and susceptible to variations, it is a necessity to mix the two samples early during this procedure. By doing this, errors caused by variations in individual sample handling are excluded thus producing a more reliable determination of the separate protein amounts. By comparing baseline and event samples in the two groups each patient serve as its own control. This approach, as compared to the more commonly used pooled sample strategy, gives a more informative picture since inter-individual variability can be assessed. Although several additional validation studies are needed, the proteins found to be regulated in the present study may be potential biomarkers for acute rejection episodes in renal transplantation. The up-regulation was detected already several days prior to the acute rejection was clinically suspected (increased creatinine). This is an interesting finding since in addition to being non-invasive the urinary proteome may hence also provide a more sensitive diagnostic approach for AR. Acute rejection episodes are a gradual processes and if the altered urine proteome turns out to be an earlier responding, and more specific, biomarker of AR it could have dramatic implications on follow-up of renal transplant recipients and their long-term outcome. It is plausible that only a minor adjustment of the immunosuppressive therapy is required to “silence” the activated immune process in an early phase, and hence avoid full activation.

In the nature of this kind of proof of principle study, sufficiently powered statistical analyses are difficult to perform. Looking at the data descriptively, only the Mannan-binding lectin serin protease 2 (MASP2) was up-regulated in all patients with AR. The extent of regulation of each protein differed individually as showed in Table 2, without any obvious pattern. In an attempt to provide a relevant statistical comparison proteins were grouped by biological function. This substantiated a potentially relevant regulation in the rejection group also of the other ten identified proteins. The data may also indicate a connection between severity of the AR and the protein regulation as the only patient with arterial changes (Banff 2 A) showed an almost universally up-regulation (10 of 11 identified proteins elevated and the last not detected). The patient who only experienced a borderline rejection further supports this hypothesis as only a relatively low degree of regulation was seen (4 of 11 proteins). The possibility to look at regulation patterns of more than one protein is an advantage of the applied method and can be crucial when looking for biomarker candidates.

For the proteins related to immune response, a strong up-regulation was observed in 4 of the AR-patients. The two remaining patients, one with only a borderline rejection actually showed a slight down-regulation. This was mostly due to a strong down-regulation of acute phase proteins Complement C3 and CD59 glycoprotein, respectively. In the control group most of the patients showed decreased levels of immune proteins, especially for the acute phase proteins.

Earlier studies present data supporting a relevant contribution of many of these proteins in AR episodes. For example, the pro-inflammatory cytokine Macrophage migration inhibitory factor (MIF), the extracellular ligand for CD74 [32,33], has been associated with AR in kidney transplants [10]. MASP2, polymeric immunoglobulin receptor, Ceruloplasmin and participants in the complement system have also been shown to be regulated in association with AR [20,34-39].

Cell growth proteins were up-regulated in five of six patients in the AR-group and are involved in several AR processes. Insulin-like growth factor-binding protein 7 (IGFBP7) modulate effects of vascular endothelial growth factor (VEGF) [40] and is reported to interact with Chemokines in venous endothelium including IFN-γ-inducible protein 10 (IP-10; CXCL10) [41,42], which has previously been reported to be elevated in urine in connection with AR [13,17]. Vasorin and Galectin-3-binding protein are closely associated to transforming growth factor-beta (TGF-*β*_1_) and IL-6, both linked to acute rejection in this population [15,43-48].

MEP1A protein does not fit to either of the two protein groups and was only detected in the AR-group, significantly up-regulated at the time of AR. The absence of identified MEP1A in the control group is an interesting observation and could potentially be very useful in a diagnostic setting. It should however be kept in mind that this is only a proof of principle study so a wide range of further validation series have to be performed to ensure that the observations are clinically relevant.

Many urinary proteins that previously have been shown to be regulated in association with AR were also detected in our study, but not regulated enough to fulfill the predefined criteria [20][49]. In addition, other proteins have been investigated using a more targeted approach (e.g. ELISA) but these were not confirmed by our investigation [10-19,21].

In addition to looking at the whole proteome a major strength of our analysis is that each patient was his or her own control, comparing the protein levels at baseline with sequential follow-up time points, in a well defined patient population. This allowed us to show that the proteins were regulated already several days before clinical suspicion of AR. In addition, individual samples were analyzed in the present study, not pooled urine, providing more detailed information of the regulation in association with the AR. The current study lack however a post treatment sample in order to be perfectly complete. With such a sample it would have proven that the regulation was specific to acute rejection episodes. Unfortunately such samples were not collected. Another indication of the relevance of the current findings is that all identified proteins are physiological plausible to be involved in an acute rejection episode. The major limitation of this proof of principle study is the relative limited small sample-size compared to classical approaches. This, however, has its cause in the time consuming and labor intensive nature of the full shotgun proteomic approach chosen. Even though it is applicable for first identification of potential biomarkers, analysis of each digested sample took almost one week to finalize. Somewhat overlap in analysis is possible but in general it is a too labor demanding procedure for large studies. In future validation of the present findings a more targeted analytical approach has to be utilized. It should also be pointed out that the control group patients were not verified non-rejectors by protocol biopsies. Previous studies have shown an incidence of almost 30% subclinical rejections in apparently stable patients on CsA based immunosuppression [50,51]. It is hence possible that sub-clinical rejections could be present in some of the controls, making the interpretation somewhat biased. Further prospective studies are needed in larger populations, where biopsies also are performed in the controls, to fully elucidate on the involvement of these proteins in AR and their potential usability as diagnostic biomarkers.

## Conclusion

This study shows the applicability of shotgun proteomics in combination with relative quantification by ^18^O/^16^O-labeling in biomarker discovery in sequential urine samples. Two groups of physiological related proteins with relevance to immunological processes during AR episodes were found to be up-regulated in patients with BPAR.

## Competing interests

The authors declare that they have no competing interests.

## Authors’ contributions

HL developed the proteomic method, designed the proteomic analysis part, performed all analyses, interpreted the results and wrote the paper. KM designed the study and the proteomic analysis part, performed the clinical trial and wrote the paper. AH designed the study, performed the clinical trial and commented on the paper. AVR performed the clinical trial and commented on the paper. PF designed the study, performed the clinical trial and commented on the paper. HH performed the clinical trial and commented on the paper. TJ performed the clinical trial and commented on the paper. LR developed the proteomic method and designed the proteomic analysis part and wrote the paper. AÅ designed the study and the proteomic analysis part, performed the clinical trial and wrote the paper. All authors read and approved the final manuscript.
